# An Optimum Design of a Subsonic Aircraft Wing due to the Aerodynamic Loading

**DOI:** 10.1155/2024/8867496

**Published:** 2024-01-17

**Authors:** Ibtisam J. Ismeal, Mehmet Bakirci, Muhsin J. Jweeg

**Affiliations:** ^1^Karabuk University, Karabuk, Türkiye; ^2^Al-Farahidi University, College of Technical Engineering, Baghdad, Iraq

## Abstract

Aircraft designers are mainly interested in finding the level of pressure, stresses, and deformations of the parts of the aircraft wing. In many aviation accidents, the failure of the wing is the main cause of disasters, as it is considered the main surface that generates the necessary lift for the aircraft in addition to its other functions in controlling the transverse stability. In this work, a numerical study was performed to obtain the optimum wing structural design parameters for high strength and minimum weight for the L-39 A/C wing. The wing was modeled as a honeycomb with different thicknesses using the software SOLIDWORKS 2020. The pressure distribution was predicted using the FLUENT 2022 R1 package. Having obtained the aerodynamic pressure, the deformations and stresses were obtained using the ANSYS program. The results were compared with other researchers using other models, such as using ribs and stringers in the interior structure of the wing. The current results were found to be reliable and acceptable from the design point of view of the high stiffness-to-weight ratio.

## 1. Introduction

The design of the internal structure of the aircraft wing mainly depends on the use of ribs and stringers, and the wing structure is divided into many cells which results in a low stiffness-to-weight ratio. The new aeroplane design trends are focused on the use of lightweight sections which are capable of supporting the payload and aerodynamic loading, such as the honeycomb structures. The authors of [1–3] presented an experimental and computational study of the bending behaviors regarding honeycomb sandwich panels with different shapes of the cores (in other words, circular, hexagonal, and square) and 2 types of facings: one is aluminum and the other is composite. In comparison with the other core shapes, the square honeycomb core had the highest load, which increased due to the increase in facing thickness, and aluminum skin facing had a larger value of the load when compared to composite skin facing.

Crushing behavior related to a honeycomb sandwich structure was investigated via experiments. In addition, a numerical model for capturing some specific deformation and failure features in the process of crushing was developed using experimental data to ensure its validity. A three-point bending test was performed on an Al honeycomb sandwich panel [4]. The strength of the sandwich structures under bending loads with a variety of face materials was studied theoretically. Titanium and aluminum may be used as face materials. It has been discovered that titanium alloy has superior sandwich construction qualities [5–7].

The bending stress of a glass fiber-reinforced plastic (GFRP) sandwich construction was investigated for a lightweight vehicle. There have been 3 different adhesives used to adhere to the face and core. The research demonstrated that a lightweight chassis vehicle's honeycomb sandwich panel design with three adhesives might withstand considerable bending stress [8–10]. The researchers provided an experimental investigation on honeycomb sandwich panel compression properties in relation to different design parameters such as cell size, foil thickness, and size of the sample (i.e., width, length, and height dimensions) regarding the honeycomb structure. Sandwich samples were constructed by bonding Al honeycomb cores and 1 mm thick CFRP laminate faces, and compression tests were performed on them. It could be seen that when the foil thickness and core height decrease, the yield stress increases [11, 12].

The impact of the honeycomb thickness on the vibration response of sandwich panels was studied using experiments with various boundary conditions. Free vibration analysis was performed on different support conditions. It was revealed that the impact of core height on the basic natural frequency of the honeycomb sandwich panels is considerable. As the height of the core rises, so does frequency [13–15].

Finite and experimental element analytic methods have been used to look at the behavior of aluminum honeycomb structures under the low-speed impacts. The ASTM D7766 standard was utilized for conducting low-velocity impact tests on the honeycomb structures that were created. The impact force was investigated as a function of cell width and height. The maximal impact force values in the honeycomb composite constructions have been found to grow as cell width and height decrease. The experimental and finite element approach results are roughly 85 percent in agreement [16–18].

The goal of the current work is to design and analyze a lightweight L-39 aircraft wing using a honeycomb structure which can resist aerodynamic loading. A comparison study will be performed by using wing design with ribs and stringers.

## 2. Honeycomb Structures

**Figure 1:**
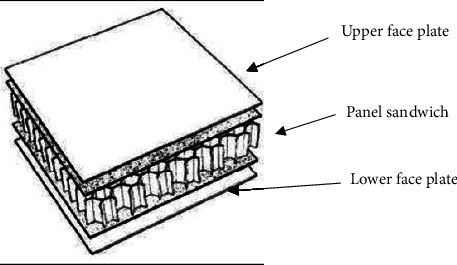
Schematic of a typical sandwich structure.

With regard to sandwich structures, honeycomb cores are available in a range of materials, including paper and card for applications needing low strength and stiffness and low loads (like interior doors for homes) and high strength and stiffness, incredibly lightweight sections for aviation structures, are available for honeycomb cores used in sandwich structures. Honeycombs can be formed into composite structures which are both flat and curved without needing a lot of mechanical force or heat [19–21]. A honeycomb's typical shape is shown in Figure 1. The cells could be hexagonal, triangular, or square. Examples of honeycomb include glass fiber-reinforced plastic, aluminum, and honeycomb made of kraft paper. Aluminum and carbon honeycomb cores were used in this experiment. Figure 1 shows a simplified honeycomb.

**Figure 2:**
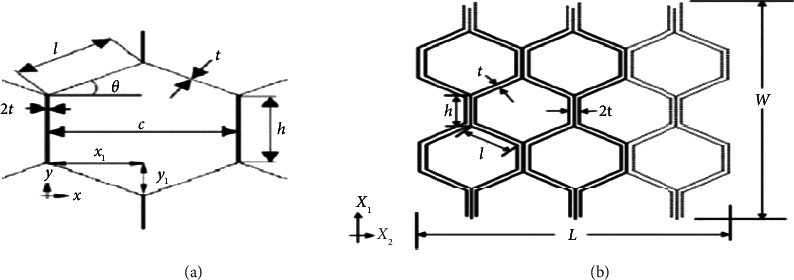
Definitions of parameters for a honeycomb cell [11]. (a) One honeycomb cell. (b) Multihoneycomb cell.

Honeycomb characteristics are anisotropic, meaning that they differ between out-plane and in-plane strengths and stiffnesses. The walls of the cells first bend, and deformation is linear elastic in a case where a honeycomb is squeezed in plane, that is, when stress acts orthogonal to the cell axis; plane X1 X2 is depicted in Figure 2.

Composite sandwich structures, on the other hand, represent a modeling problem due to the fact that their core region is made up of several cells with complex geometries.

**Figure 3:**
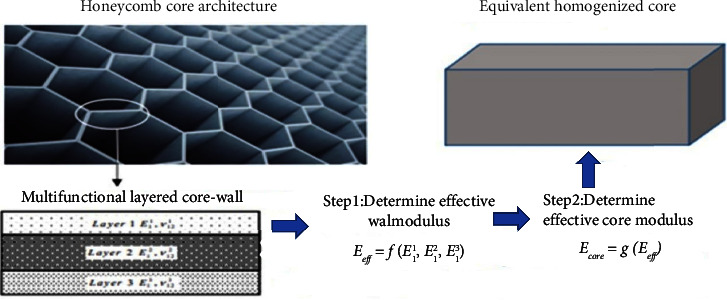
Overview of the homogenization methodology sought in the current investigation.

The isotropic beam analysis method in [22–25] is the most widely used model of unit cells for determining effective characteristic determination. The author makes an assumption that the honeycomb deformations' linear elastic response and the consequent core characteristics are solely dependent on the bending of the core cell walls. The stretching and shearing of cell walls were studied as additional deformation modes [26–28]. FEM has been used in numerous previous publications for estimating effective material characteristics regarding honeycomb architectures. The core of multifunctional sandwich composites could then be sized using analytical equations in the design process to account for anticipated energy needs as well as desired service loads.  Figure 3 provides an illustration of the homogenization approach used in this study.

In Figure 3, the superscript in *E*_1_^1^, *E*_1_^2^, and *E*_1_^3^ refers to the layer number and the subscript refers to the principal direction. *E*_ff_ is the effective modulus, and *E*_core_ is the core modulus.

### 2.1. Material Properties of the Sandwich Panels

The material properties used in this analysis for carbon fibers and Al 7075-T6 materials are listed in Table 1. A 3D deformable shell geometry is used to model the skins and the honeycomb core. The material properties for aluminum (Al 7075-T6) are as follows: elastic modulus = 72 000 N/mm^2^, Poisson's ratio = 0.3, shear modulus = 26900 N/mm^2^, tensile strength = 570 N/mm^2^, yield strength = 505 N/mm^2^, mass density = 2810 kg/m^3^, thermal expansion coefficient = 2.36*E*.05 1/K, thermal conductivity = 130 W/(m·K), and specific heat = 960 J/(kg·K).

Material properties of carbon fiber laminates are *E* = 140 GPa, density = 1760 kg/m^3^, and Poisson's ratio = 0.22.

**Figure 4:**
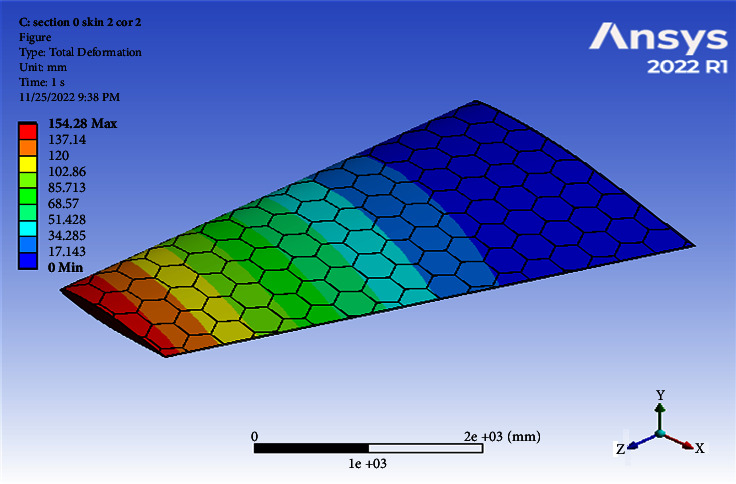
Contour of the total deformation at one cell skin 2 core 2 (Mach 0.4).

The work methodology is shown as block diagram in Figure 4, which can be summarized as follows: (1) Three internal structure configurations were suggested: 1 cell, 6 cells, and 9 cells. (2) For each case in (1), three different air speeds were chosen 0.4, 0.6, and 0.8 Mach numbers. (3) The aerodynamic pressure distribution on the wing for each case in (2) is obtained using the FLUENT program. (4) For each case in (1), the skin thicknesses of 2, 3, and 4 mm are used. (5) For each skin thickness in (4), three core thicknesses of 2, 4, and 6 mm are used. (6) The displacement and stresses are obtained for each tested case in the block diagram using the ANSYS package.

## 3. Detailed Case Study

The L-39 A/W wing weight is 6500 kg, with the profile NACA 64A 012. The span is 9.12 m, the gross area is 18.8 m^2^, the quarter chord line sweepback angle is 1°45, the leading edge sweepback angle is 6°26, the taper ratio is 0.475, the aspect ratio is 4.4, the mean aerodynamic chord is 2.15 m, the tip chord is 1.33 m, the root chord is 2.8 m, and the geometric shape is trapezoidal.

The calculations are based on the angle of attack 12° because of the maximum pressure around the A/C wing model as shown in Table 1. First, a 12° angle of attack is adopted because at this attitude, the maximum deformations and stresses are developed in the wing structure. The pressure developed at this position is found using the FLUENT package which is exported directly to the ANSYS package (fluid-structure interaction) which is considered an exact modeling of the wing structure under aerodynamic modeling. This was used throughout the work presented here.

### 3.1. Wing One Cell

The determination of displacements and stress using different structural modeling methods is presented here. Finite element modeling was employed as explained above to predict the pressure distribution to be applied on the wing structure, which corresponds to the real case to give the true picture of deformation and stresses on the wing skin.

The effects of the design parameters of the wing structure are discussed as follows:

- Effects of skin thickness
- Effects of the number of cells
- Effects of core thickness (invariably of skin thickness)

#### 3.1.1. Effects of Skin Thickness

**Figure 5:**
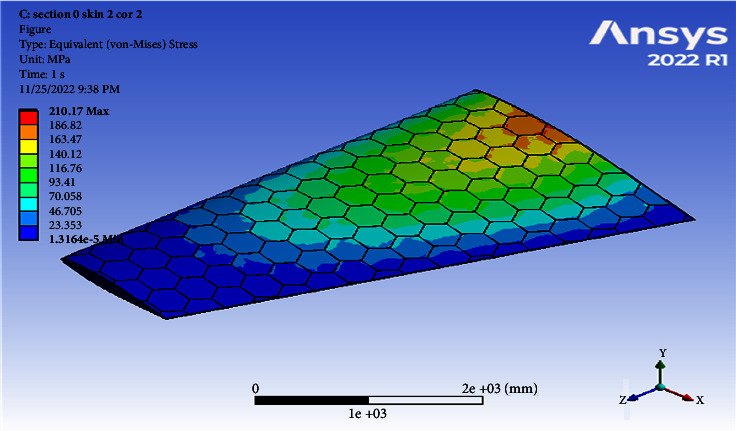
Contour of the equivalent stress at one cell skin 2 core 2 (Mach 0.4).

Using a skin thickness of 2 mm and a core thickness of 2 mm, the maximum deformation of 154.28 mm and the resulted von Mises of 210.17 MPa were obtained, as shown in Figures 4 and 5.

**Figure 6:**
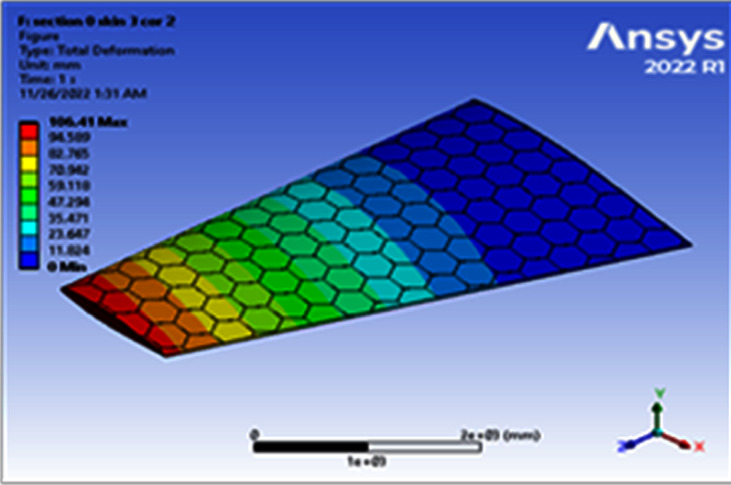
Contour of the total deformation at one cell skin 3 core 2 (Mach 0.4).

**Figure 7:**
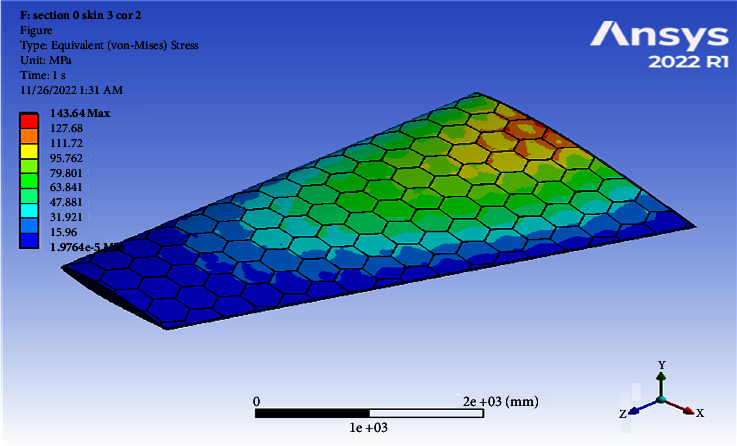
Contour of the equivalent stress at one cell skin 3 core 2 (Mach 0.4).

Increasing a skin thickness to 3 mm, the deformation becomes 106.41 mm and the resulted von Mises becomes 143.64 MPa, as shown in Figures 6 and 7.  Table 2 shows the results of using different thicknesses (invariably of core). Note that in Table 2, some of the points in bold failed because the value of the von Mises stress exceeded a yield strength of 505 N/(mm)^2^. The least mass was 200.21 kg and the largest mass was 421.71 kg using material Al 7075-T6.  Table 2 shows that the change in the total von Mises stress was largest (793.92 MPa) at a skin thickness of 2 mm, a core thickness of 4 mm, and Mach 0.8 (failed), while the lowest stress (108.41 MPa) was obtained at a skin thickness of 4 mm, a core thickness of 6 mm, and Mach 0.4. It was shown that the largest equivalent strain of 0.0075414 mm/mm was obtained in the model with a skin thickness of 3 mm and a core thickness of 2 mm at Mach 0.8, while the least deviation of 0.0010246 mm/mm was obtained in the model with a skin thickness of 2 mm and a core thickness of 6 mm at Mach 0.8.

#### 3.1.2. Effects of Core Thickness

Using a skin thickness of 2 mm and a core thickness of 2 mm, the maximum deformation of 338.84 mm and the resulted von Mises of 473.87 MPa were obtained, as shown in Table 3.

Note that in Table 3, some of the points in bold failed because the value of the von Mises stress exceeded a yield strength of 505 N/mm^2^. The least mass was 200.21 kg, and the largest mass was 421.71 kg. Table 3 shows that the change in the total von Mises stress was largest (793.92 MPa) at a skin thickness of 2 mm, a core of thickness 4 mm, and Mach 0.8 (failed), while the lowest stress (108.41 MPa) was obtained at a skin thickness of 4 mm, a core thickness of 6 mm, and Mach 0.4. Table 3 also shows that the largest equivalent strain of 0.0075414 mm/mm was obtained in the model with a skin thickness of 3 mm and a core thickness of 2 mm at Mach 0.8, while the least deviation of 0.0010246 mm/mm was obtained in the model with a skin thickness of 2 mm and a core thickness of 6 mm at Mach 0.8. Some cases failed due to the maximum stresses developed compared to the yield stress at the used material (Al 7075-T6) when a stress ratio is less than 1. They are labelled in bold. Even the masses are low compared to the mass used in the A/C design.

### 3.2. Wing 6 Cells

Finite element modeling was used as stated previously to predict the pressure distribution to be applied on the wing structure. This corresponds to the real case to give the true picture of deformation and stresses on the wing skin. The effects of the design parameters of the wing structure are discussed as follows:

- Effects of skin thickness.
- Effects of the number of cells
- Effects of core thickness (invariably of skin thickness)

#### 3.2.1. Effects of Skin Thickness

**Figure 8:**
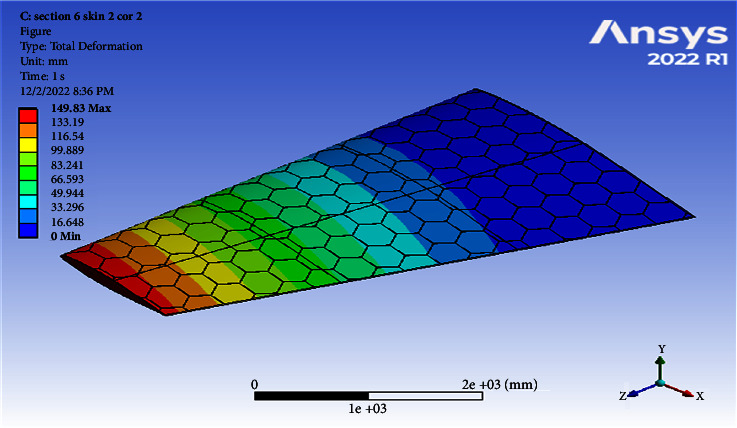
Contour of the total deformation at 6 cell skin 2 core 2 (Mach 0.4).

**Figure 9:**
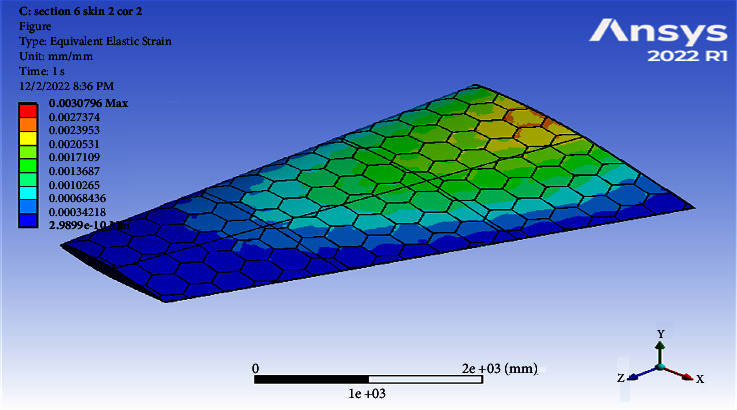
Contour of the equivalent stress at 6 cell skin 2 core 2 (Mach 0.4).

**Figure 10:**
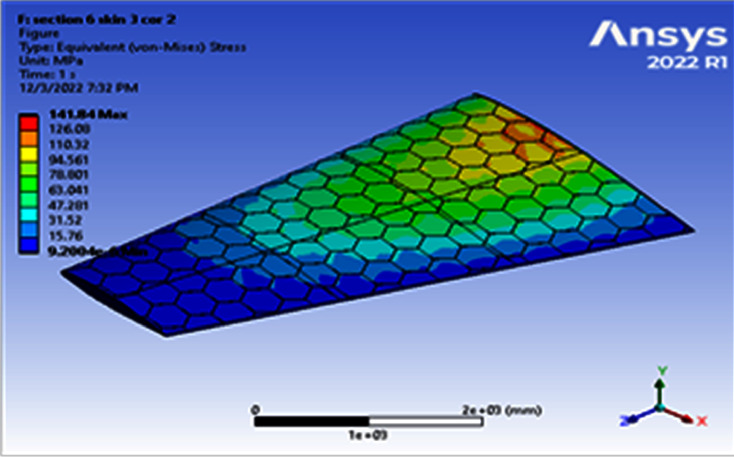
Contour of the equivalent stress at 6 cell skin 3 core 2 (Mach 0.4).

**Figure 11:**
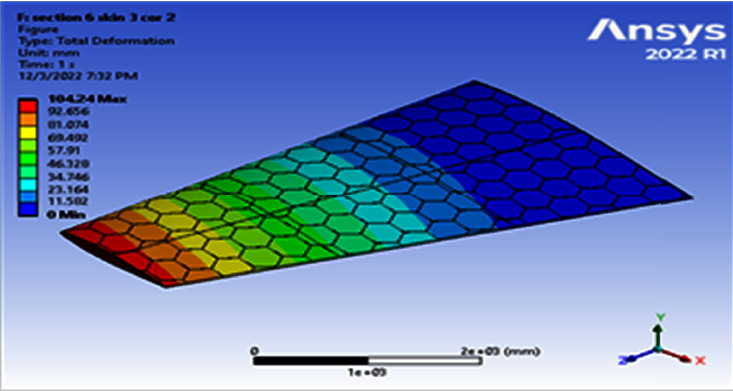
Contour of the total deformation at 6 cell skin 3 core 2 (Mach 0.4).

**Figure 12:**
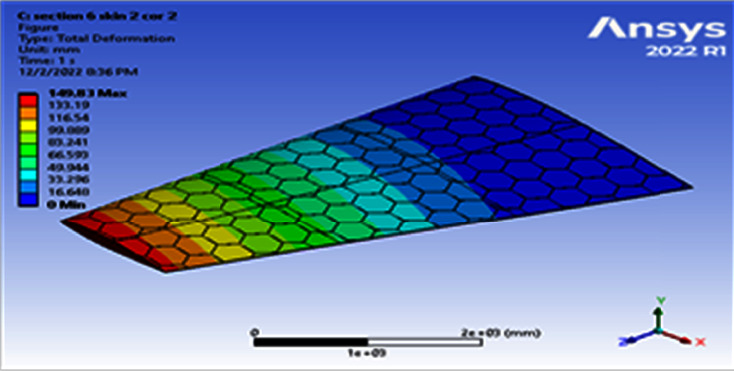
Contour of the total deformation at 6 cell skin 2 core 2 (Mach 0.6).

**Figure 13:**
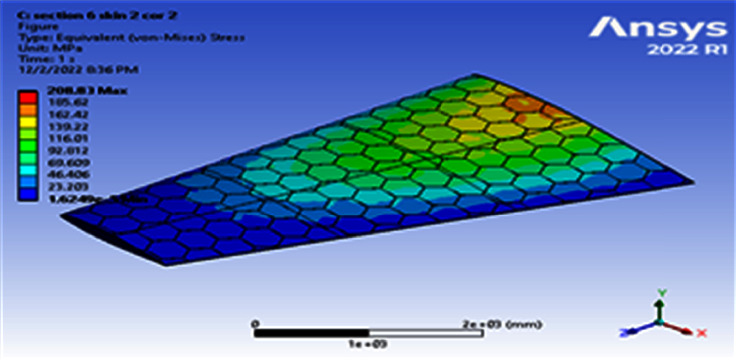
Contour of the equivalent stress at 6 cell skin 2 core 2 (Mach 0.6).

Using a skin thickness of 2 mm and a core thickness of 2 mm, the maximum deformation of 149.83 mm and the resulted von Mises of 208.83 MPa were obtained, as shown in Figures 8–11. Increasing the skin thickness to 3 mm, the deformation becomes 104.24 mm and the resulted von Mises becomes 141.84 MPa, as shown in Figures 12 and 13.

Table 4 shows the results of using different thicknesses (invariably of core). Note that in the table, some of the points in bold failed because the value of the von Mises stress exceeded a yield strength of 505 N/(mm)^2^. The least mass was 210.46 kg and the largest mass was 451.26 kg using material Al 7075-T6.  Table 5 shows that the change in the total von Mises stress was largest (765.07 MPa) at a skin thickness of 2 mm, a core thickness of 2 mm, and Mach 0.8 (failed), while the lowest stress (100.77 MPa) was obtained at a skin thickness of 4 mm, a core thickness of 6 mm, and Mach 0.4. This table also shows that the largest equivalent strain of 0.0011286 mm/mm occurs in the model with a skin thickness of 4 mm and a core thickness of 6 mm at Mach 0.8, while the least deviation is 0.001417 mm/mm obtained in the model with a skin thickness of 2 mm and a core thickness of 6 mm at Mach 0.4.

#### 3.2.2. Effects of Core Thickness

**Figure 14:**
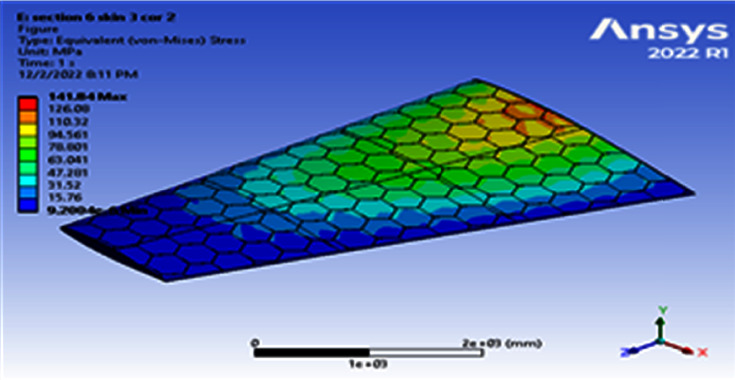
Contour of the equivalent stress at 6 cell skin 3 core 2 (Mach 0.6).

**Figure 15:**
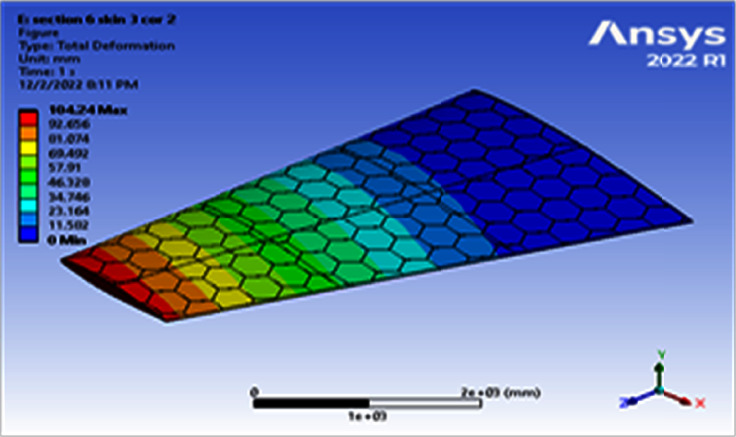
Contour of the total deformation at 6 cell skin 3 core 2 (Mach 0.6).

Using a skin thickness of 2 mm and a core thickness of 2 mm, the maximum deformation is 149.83 mm and the resulted von Mises is 208.83 MPa. Increasing a skin thickness of 3 mm, the deformation is 104.24 mm and the resulted von Mises is 141.84 MPa, as shown in Figures 12–15.

Table 5 shows the results of using different thicknesses (invariably of core). Note that in the table, some of the points in bold failed because the value of the von Mises stress exceeded yield strength = 505 N/(mm)^2^. The least mass was 210.46 kg and the largest mass was 451.26 kg using material Al 7075-T6.  Table 5 also shows that the change in the total von Mises stress was largest (765.07 MPa) at a skin thickness of 2 mm, a core thickness of 2 mm, and Mach 0.8 (failed), while the lowest stress (100.77 MPa) was obtained at a skin thickness of 4 mm, a core thickness of 6 mm, and Mach 0.4. This table also shows that the largest equivalent strain of 0.0011286 mm/mm occurs in the model with a skin thickness of 4 mm and a core thickness of 6 mm at Mach 0.8, while the least deviation of 0.001417 mm/mm was obtained in the model with a skin thickness of 2 mm and a core thickness of 6 mm at Mach 0.4.

The summary of the results which are extracted from the contours is shown in Table 5. Some cases failed due to the maximum stresses developed compared to the yield stress at the used material (Al 7075-T6) when a stress ratio is less than 1. They are labelled in bold.

## 4. Comparison Study

The obtained results using the honeycomb structures are compared with the results obtained by using the ribs and stringers as shown in Table 6.

Table 6 shows a comparison study between the suggested modeling and the work achieved by Gargan [29].

## 5. Conclusions

From this work, the following points can be concluded:

1. The effectiveness of using honeycomb in reducing stress is greater than the effectiveness of using the ribs and stringers.
2. Using 7075-T6 aluminum alloy for both the skin and core gives the least weight of the wing structure with a largest weight (421.71 kg) using one cell and a minimum weight using 6 cells (200.2 kg). The maximum wing mass was 451.26 kg and the minimum mass was 210.46 kg which are considered to be lightweight wings in any case compared to the wings with ribs and stringers with a minimum mass of 414 kg.
3. The stiffness of the wings using the honeycomb structures is greater than that using the ribs and stringers, which results in less deformation, which in turn increases the flutter and the divergence speeds and gives a greater chance to avoid failure under the same aerodynamic modeling.
4. Finally, the finite element modeling of a three-dimensional wing and using the idea of cells as a stiffening element were found to give good results and to determine the areas of most stress and acceptable deformation levels.

## Figures and Tables

**Table 1:** Work plan.

Displacements and von Mises stresses																											
	1 cell									6 cells									9 cells								
Mach no.	*M* = 0.4									*M* = 0.4									*M* = 0.4								

Skin thickness (mm)	2			3			4			2			3			4			2			3			4		
Core thickness (mm)	2	4	6	2	4	6	2	4	6	2	4	6	2	4	6	2	4	6	2	4	6	2	4	6	2	4	6

Mach no.	*M* = 0.6									*M* = 0.6									*M* = 0.6								

Skin thickness (mm)	2			3			4			2			3			4			2			3			4		
Core thickness (mm)	2	4	6	2	4	6	2	4	6	2	4	6	2	4	6	2	4	6	2	4	6	2	4	6	2	4	6

Mach no.	*M* = 0.8									*M* = 0.8									*M* = 0.8								

Skin thickness (mm)	2			3			4			2			3			4			2			3			4		
Core thickness (mm)	2	4	6	2	4	6	2	4	6	2	4	6	2	4	6	2	4	6	2	4	6	2	4	6	2	4	6

**Table 2:** Wing one cell at angle of attack 12° (2 cells).

Mach no.	Skin thickness (mm)	Core thickness (mm)	Max deformation (mm)	Equivalent stress (MPa)	Equivalent strain (mm/mm)	Mass (kg)
0.4	2	2	154.28	210.17	0.003177	200.21
	3	2	106.41	143.64	20404	258.8
	4	2	81.681	110.74	0.001557	317.96
	2	4	146.39	217.08	0.003156	253.46
	3	4	101.88	140.54	0.002052	311.5
	4	4	78.785	108.41	0.001577	369.96
	2	6	138.16	195.19	0.002785	306.56
	3	6	98.262	137.65	0.001964	363.82
	4	6	76.48	104.37	0.001484	421.71

0.6	2	2	338.84	473.87	0.007152	200.1
	3	2	233.76	324.6	0.004612	258.8
	4	2	179.45	253.16	0.003528	317.96
	2	4	321.44	487.8	0.007093	253.46
	3	4	223.74	316	0.004617	311.5
	4	4	173.04	244.05	0.003549	369.96
	2	6	308.4	515.52	0.007238	306.56
	3	6	215.75	309.67	0.004423	363.82
	4	6	167.95	235.52	0.003338	421.71

0.8	2	2	547.06	774.53	0.011689	200.1
	3	2	377.45	530.72	0.007541	258.8
	4	2	289.77	413.8	0.005768	317.96
	2	4	518.87	793.92	0.011543	253.46
	3	4	361.19	514.4	0.007548	311.5
	4	4	279.36	398.98	0.005801	369.96
	2	6	489.42	718.16	0.010246	306.56
	3	6	348.26	503.78	0.007197	363.82
	4	6	271.12	358.11	0.005433	421.71

**Table 3:** Wing one cell at angle of attack 12° (6 cells).

Mach no.	Skin thickness (mm)	Core thickness (mm)	Max deformation (mm)	von Mises stress (MPa)	Equivalent strain (mm/mm)	Mass (kg)
0.4	2	2	154.28	210.17	0.003177	200.2
	2	4	146.39	217.08	0.003156	253.5
	2	6	138.16	195.19	0.002785	306.6
	3	2	106.41	143.64	0.00204	258.8
	3	4	101.88	140.54	0.002052	311.5
	3	6	98.262	137.65	0.001964	363.8
	4	2	81.681	110.74	0.001557	318
	4	4	78.785	108.41	0.001577	370
	4	6	76.48	104.37	0.001484	421.7

0.6	2	2	338.84	473.87	0.00715	200.2
	2	4	321.44	487.8	0.00709	253.5
	2	6	308.4	515.52	0.007238	306.6
	3	2	233.76	324.6	0.00461	258.8
	3	4	223.74	316	0.00462	311.5
	3	6	215.75	309.67	0.00442	363.8
	4	2	179.45	253.16	0.00353	318
	4	4	173.04	244.05	0.00355	370
	4	6	167.95	235.52	0.00334	421.7

0.8	2	2	547.06	774.53	0.011689	200.21
	2	4	518.87	793.92	0.011543	253.46
	2	6	489.42	718.16	0.010246	306.56
	3	2	377.45	530.72	0.007541	258.8
	3	4	361.19	514.4	0.007548	311.5
	3	6	348.26	503.78	0.007197	363.82
	4	2	289.77	413.8	0.005768	317.96
	4	4	279.36	398.98	0.005801	369.96
	4	6	271.12	358.11	0.005433	421.71

**Table 4:** Wing 6 cells at the angle of attack 12°.

Mach no.	Skin thickness (mm)	Core thickness (mm)	Max deformation (mm)	von Mises stress (MPa)	Equivalent strain (mm/mm)	Mass (kg)
0.4	2	2	149.83	208.83	0.00308	210.46
	3	2	104.24	141.84	0.002064	268.93
	4	2	80.478	113.23	0.001575	328.02
	2	4	138.66	203.51	0.002887	273.77
	3	4	98.149	136.93	0.001962	331.6
	4	4	76.558	104.97	0.001509	389.87
	2	6	129.37	196.4	0.002781	336.7
	3	6	93.145	130.62	0.001839	393.69
	4	6	73.405	100.77	0.001417	451.26

0.6	2	2	329.04	467.96	0.0069023	210.46
	3	2	228.96	321.24	0.0046238	268.93
	4	2	176.79	260.86	0.003631	328.02
	2	4	304.44	456.06	0.0064775	273.77
	3	4	215.53	307.49	0.0044095	331.6
	4	4	168.13	237.86	0.003391	389.87
	2	6	283.99	441.37	0.0065513	336.7
	3	6	204.5	294.99	0.004155	393.69
	4	6	161.18	227.4	0.003184	451.26

0.8	2	2	531.3	765.07	0.011286	210.46
	3	2	369.72	526.43	0.007557	268.93
	4	2	285.5	426.58	0.005938	328.02
	2	4	491.52	746.52	0.010595	273.77
	3	4	347.99	504.07	0.00723	331.6
	4	4	271.47	389.03	0.005558	389.87
	2	6	458.47	724.37	0.010264	336.7
	3	6	33.07	480.62	0.006771	393.69
	4	6	260.24	372.02	0.00522	451.26

**Table 5:** The results of using different thicknesses (invariably of core).

Mach no.	Skin thickness (mm)	Core thickness (mm)	Max deformation (mm)	Equivalent stress (MPa)	Equivalent strain (mm/mm)	Mass (kg)
0.4	2	2	149.83	208.83	0.00308	210.46
	2	4	138.66	203.51	0.002887	273.77
	2	6	129.37	196.4	0.002781	336.7
	3	2	104.24	141.84	0.002064	268.93
	3	4	98.149	136.93	0.001962	331.6
	3	6	93.145	130.62	0.001839	393.69
	4	2	80.478	113.23	0.001575	328.02
	4	4	76.558	104.97	0.001509	389.87
	4	6	73.405	100.77	0.001417	451.26

0.6	2	2	329.04	467.96	0.0069023	210.46
	2	4	304.44	456.06	0.0064775	273.77
	2	6	283.99	441.37	0.0065513	336.7
	3	2	228.96	321.24	0.0046238	268.93
	3	4	215.53	307.49	0.0044095	331.6
	3	6	204.5	294.99	0.004155	393.69
	4	2	176.79	260.86	0.003631	328.02
	4	4	168.13	237.86	0.003391	389.87
	4	6	161.18	227.4	0.003184	451.26

0.8	2	2	531.3	765.07	0.011286	210.46
	2	4	491.52	746.52	0.010595	273.77
	2	6	458.47	724.37	0.010264	336.7
	3	2	369.72	526.43	0.007557	268.93
	3	4	347.99	504.07	0.00723	331.6
	3	6	330.17	480.62	0.006771	393.69
	4	2	285.5	426.58	0.005938	328.02
	4	4	271.47	389.03	0.005558	389.87
	4	6	260.24	372.02	0.00522	451.26

**Table 6:** Comparison between the current work and that reported by Gargan [29].

Current work					
Skin thickness (mm)	Core thickness (mm)	Max deformation (mm)	von Mises stress (MPa)	Stress ratio	Mass (kg)
2	2	338.84	473.87	1.066	200.21
2	4	321.44	487.8	1.035	253.46
2	6	308.4	515.52	0.979	306.56
3	2	233.76	324.6	1.556	258.8
3	4	223.74	316	1.598	311.5
3	6	215.75	309.67	1.631	363.82
4	2	179.45	253.16	1.995	317.96
4	4	173.04	244.05	2.064	369.96
4	6	167.95	235.52	2.144	421.71

Mr. Gargan [29]					
Skin thickness (mm)	Displacement (m)	von Mises stress (MPa)		Mass (kg)	

15	0.056	460		414	
2	0.047	361		440	
25	0.041	298		467	
3	0.037	254		493	
3.5	0.032	221		520	

## Data Availability

No data were used to support the findings of this study.
